# Supplementary figures and images for: Microbial transfer through fecal strings on eggs affects leaf beetle microbiome dynamics
Source: mSystems. 2025 May 13;10(6):e01723-24. doi: 10.1128/msystems.01723-24 (PMC12172492; doi:10.1128/msystems.01723-24)

**Fig. S1
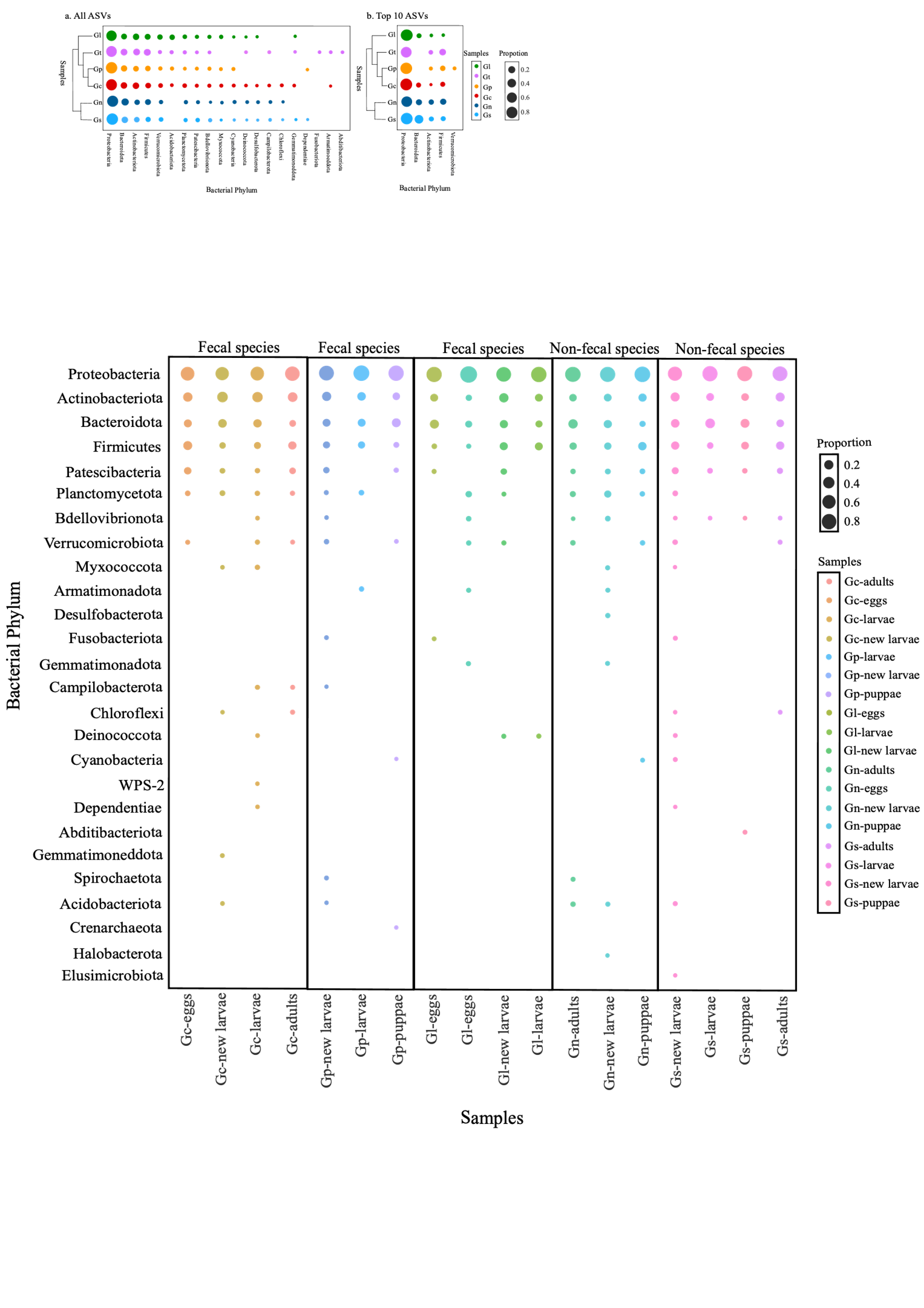
**

**Fig. S2**

**
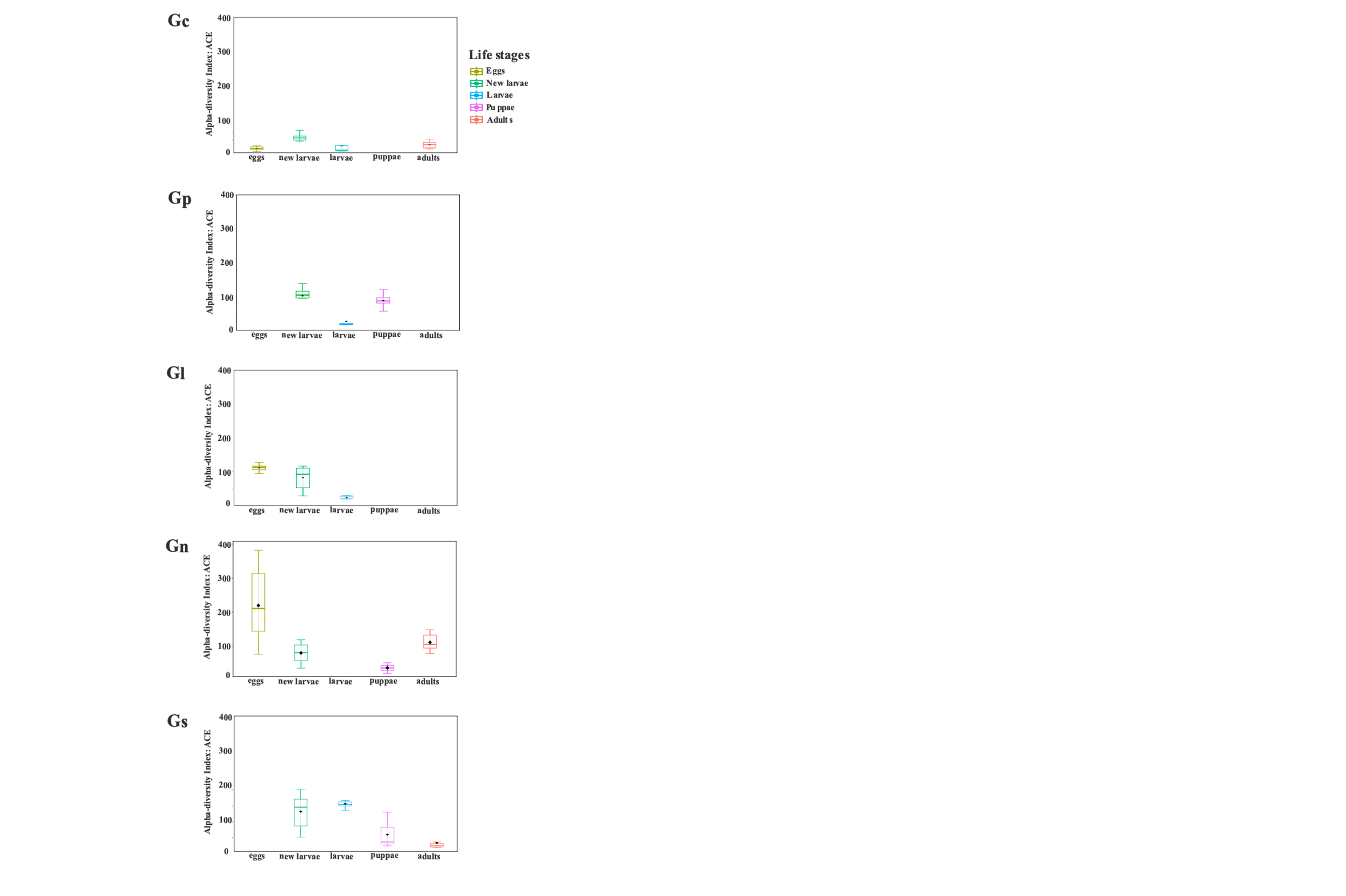
**

**Fig. S3**


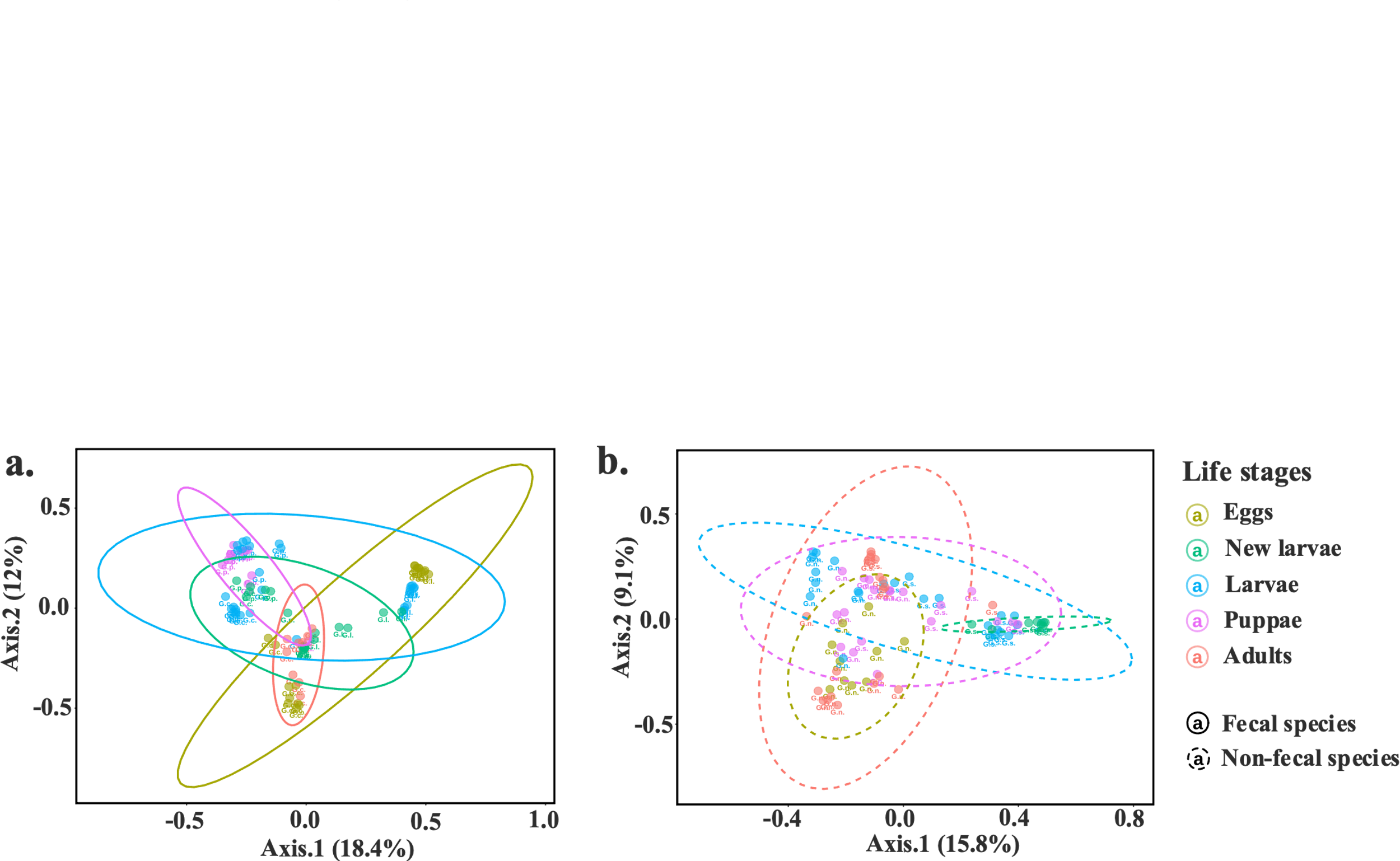


**Fig. S4**


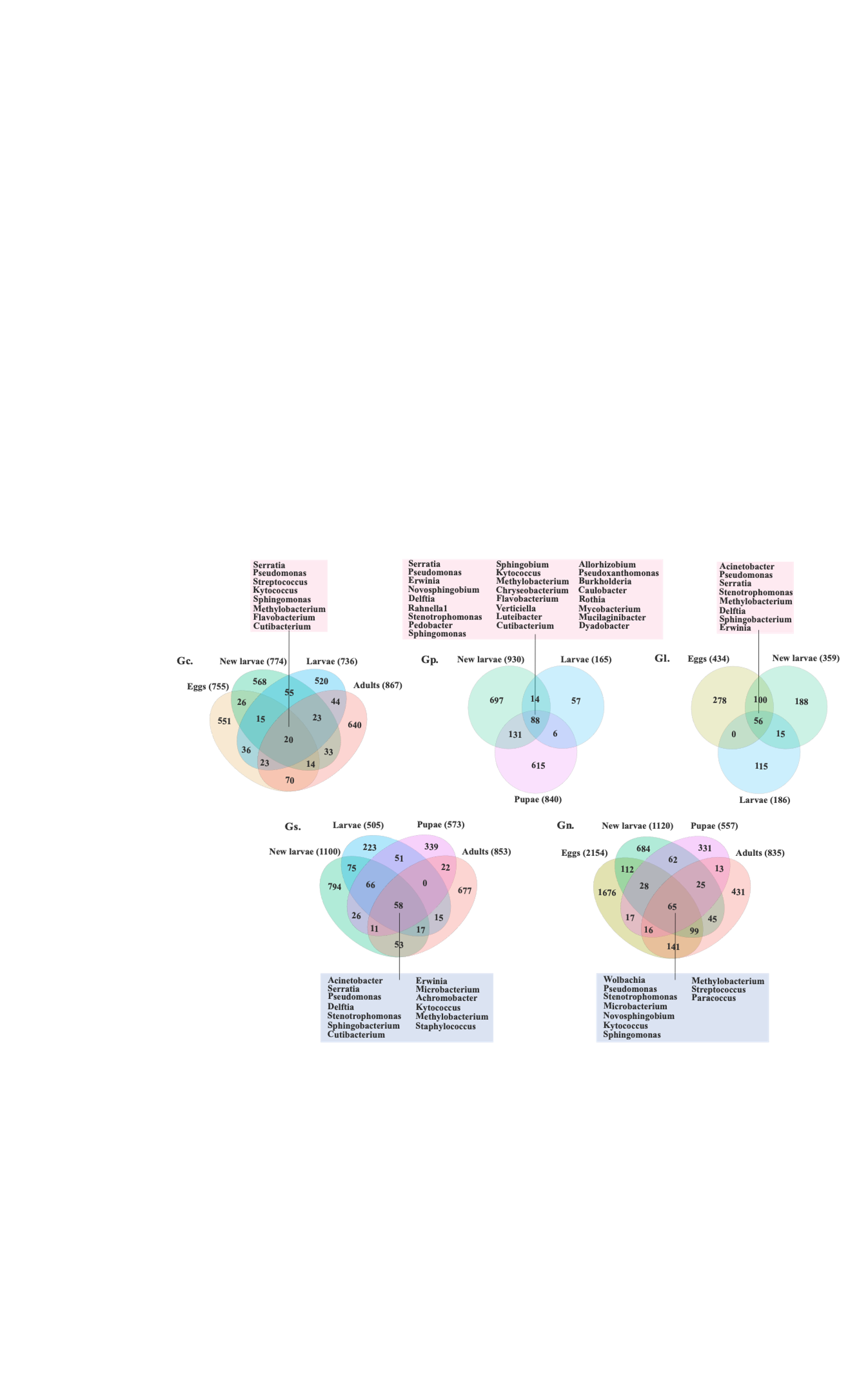

Supplement: Supplemental figures — Fig. S1 to S4. [file msystems.01723-24-s0001.docx]
