# Supplementary material for: Microbial transfer through fecal strings on eggs affects leaf beetle microbiome dynamics
Source: mSystems. 2025 May 13;10(6):e01723-24. doi: 10.1128/msystems.01723-24 (PMC12172492; doi:10.1128/msystems.01723-24)
Supplement: Legends — Supplemental figure legends. [file msystems.01723-24-s0002.docx]

**Appendix Figures**

**Fig. S1** The proportion distribution of relative abundances of bacterial communities at phylum level in different life stages of five beetle species (Gc = *Galerucella calmariensis*, Gp = *G. pusilla*, Gl = *G. lineola*, Gn = *G. nymphaeae*, Gs = *G. sagittariae*).

**Fig. S2** Boxplots of ACE index among different species (Gc = *Galerucella calmariensis*, Gp = *G. pusilla*, Gl = *G. lineola*, Gn = *G. nymphaeae*, Gs = *G. sagittariae*).

**Fig. S3** PCoA plots of Bray–Curtis distances comparing gut microbiota composition among the five life stages in the (a) fecal species and (b) non-fecal species, respectively.

**Fig. S4** Venn diagram of ASV distribution across life stages in five *Galerucella* species (Gc = *Galerucella calmariensis*, Gp = *G. pusilla*, Gl = *G. lineola*, Gn = *G. nymphaeae*, Gs = *G. sagittariae*) (ASV taxa for each species shown in table S1).
